# Research progress on the association between lung cancer and pulmonary embolism

**DOI:** 10.1186/s13019-024-03321-6

**Published:** 2025-01-03

**Authors:** Yi-Wen Zhang, Rui Tao, Dan Wu, Jin-Chun Ou, Zhuo-Chao Kong, Zhen-Zhen Zhu

**Affiliations:** Department of Respiratory Medicine, Anhui Medical University Clinical College of Chest & Anhui Chest Hospital, Hefei, 230022 People’s Republic of China

**Keywords:** Concurrent onset time, Lung cancer, Mechanism differences, Prognosis, Pulmonary embolism

## Abstract

Pulmonary embolism (PE), a form of venous thromboembolism, is a frequently observed complication in malignancies, with a notably high incidence in individuals with lung cancer. The presence of PE markedly reduces the quality of life and has a significant impact on the prognosis of those diagnosed with both lung cancer and PE. As a result, timely diagnosis and intervention are of paramount importance. The presence of PE markedly reduces the quality of life and has a significant impact on the prognosis of those diagnosed with both lung cancer and PE. As a result, timely diagnosis and intervention are of paramount importance.

## Background

Lung cancer is the second most prevalent malignancy globally and remains the leading cause of cancer-related mortality [1]. In 2020, lung cancer was responsible for 1.8 million deaths, accounting for 18% of all cancer-related fatalities [1, 2]. Pulmonary embolism (PE), a serious complication in individuals with lung cancer, is the second most common cause of death in this population, following disease progression. The incidence of PE in individuals with lung cancer is approximately six times higher than in the general population [3], placing lung cancer among the malignancies with the highest rates of thrombosis [4]. This malignancy accounts for the largest absolute number of venous thromboembolism (VTE) events associated with cancer. Factors such as high D-dimer levels, chemotherapy, deep venous thrombosis (DVT), advanced disease stages (III–IV), and adenocarcinoma have been positively correlated with the occurrence of PE. Additionally, PE is consistently associated with a poor prognosis in individuals with lung cancer [5].

The prognosis for those with coexisting lung cancer and PE is generally unfavorable, with a significant incidence rate in this patient group. Clinically, the development of PE requires close consideration, as variations in the timing of onset—whether before, during, or after the diagnosis of lung cancer—present distinct differences in pathogenesis. These variations can complicate the diagnostic and therapeutic approach. During anti-tumor treatment, lung cancer may exacerbate pre-existing PE, and when combined with infection or tumor progression, it remains uncertain whether anti-tumor therapies can lead to the chronic progression of acute PE or its conversion to chronic pulmonary hypertension.

Furthermore, the management of individuals undergoing anti-tumor therapy for lung cancer is complicated by the need to balance increased risks of both thrombosis and hemorrhage. The complex interaction between physiological responses and pharmacological interventions poses additional challenges in clinical management. This study aims to investigate the relationship between lung cancer and PE, review current research findings, and discuss the implications for clinical practice.

## Incidence and prognostic implications of PE as an early indicator of malignancy

In the absence of identifiable risk factors, a significant proportion of individuals diagnosed with PE are subsequently found to have malignancies within one year [6]. Unexplained PE may thus serve as an early marker of malignant tumors. A study by Li et al. reported that 6 patients experienced PE within 6 months prior to their cancer diagnosis [7]. Similarly, in a study by Cui et al., 30 patients were diagnosed with cancer during hospitalization for PE [8]. Moreover, 47 patients were diagnosed with PE following the identification of a malignancy, while 18 individuals experienced PE prior to their cancer diagnosis. Among these 18 cases, 3 developed PE within 3 months of the cancer diagnosis, 5 between 3 and 6 months, and 10 more than 6 months before tumor detection.

The 2018 Chinese guidelines for PE emphasize the importance of investigating the underlying cause during the diagnostic and therapeutic process [9]. In cases of VTE without an identifiable cause, close follow-up is recommended. If rheumatic immunological diseases and myeloproliferative disorders are excluded, heightened vigilance for potential malignancies is advised. Malignancies, as a prominent acquired risk factor, can induce a hypercoagulable state. In cases where tumor symptoms are not evident, this hypercoagulability may be the initial manifestation, particularly when persistent.

For the management of acute PE, a 3 month course of therapy is generally recommended following successful initial treatment. However, after discontinuation of anticoagulation therapy, secondary recurrence may occur. In a follow-up study of individuals with acute PE, those with secondary recurrences were observed to develop lung malignancies within one to three years of continued monitoring [10].

The occurrence of PE prior to a lung cancer diagnosis presents a significant clinical risk. Maintaining vigilance and ensuring thorough follow-up in cases of refractory PE can facilitate the early detection of lung cancer, allowing for prompt diagnosis and intervention, which may improve patient outcomes.

## Complex interrelationship and diagnostic challenges of pulmonary embolism in lung cancer

As the understanding of the relationship between PE and lung cancer deepens, the complex clinical relationship between these two conditions have become more apparent. Advanced computed tomography (CT) scans conducted for suspected lung cancer frequently reveal the concurrent presence of PE. Conversely, computed tomography pulmonary angiography (CTPA) performed for suspected PE occasionally identifies underlying lung cancer as the cause.

In one study, 30 patients (31.6%) were diagnosed with malignant tumors concurrently with PE during hospitalization, representing the largest proportion of such cases. [8] However, literature addressing the simultaneous diagnosis of lung cancer and PE remains limited. Many individuals with lung cancer complicated by PE do not present with distinct clinical symptoms. Common symptoms, such as hemoptysis and cough, do not significantly differ between those diagnosed solely with lung cancer and those with both lung cancer and PE.

A study by Chuang et al., involving 24 patients with both lung cancer and concurrent PE, found no statistically significant differences in the incidence of dyspnea, hemoptysis, cough, or chest pain between these patient groups [11]. Similarly, another study, comparing 28 patients with both lung cancer and PE to a control group of 56 patients with only lung cancer, identified a 7.1% prevalence of PE in the lung cancer-only cohort, without significant differences in symptoms such as cough and chest pain between the two groups [12].

The diagnostic approach to lung cancer complicated by PE relies heavily on the measurement of D-dimer levels. D-dimer, a degradation product of cross-linked fibrin produced during fibrinolysis, serves as a sensitive marker for thrombotic events or hypercoagulability. Its utility lies in its high negative predictive value for PE; a D-dimer level below 0.5 mg/ml is often sufficient to rule out PE [13]. In individuals with lung cancer and suspected PE, CTPA has proven to be highly reliable and safe, offering both diagnostic confirmation and precise localization of thrombotic lesions. [14] Individuals with lung cancer presenting with elevated D-dimer levels and a Wells score greater than two should be prioritized for CTPA, as it is an effective diagnostic tool for detecting PE.

In addition, electrocardiogram (ECG) findings, such as right ventricular hypertrophy, and the number of ECG abnormalities may help predict the likelihood of PE involving the pulmonary trunk and main pulmonary artery (MPA) [15]. The Wells score, the most commonly used pre-test probability indicator for PE in the UK, evaluates an individual's risk of PE based on clinical factors [16]. An RV/LV ratio greater than one, determined via echocardiography or CT, indicates a higher risk of adverse outcomes. Furthermore, elevated levels of serum biomarkers, such as Troponin I or T (indicating myocardial ischemia) and NT-proBNP or proNBNP (indicating myocardial stretch), suggest right ventricular dysfunction and an increased risk of hemodynamic instability. In cases where CTPA is contraindicated, such as iodine allergy or renal impairment, contrast-enhanced magnetic resonance angiography (MRA) provides a viable alternative for imaging the pulmonary vasculature.

**Fig. 1:**
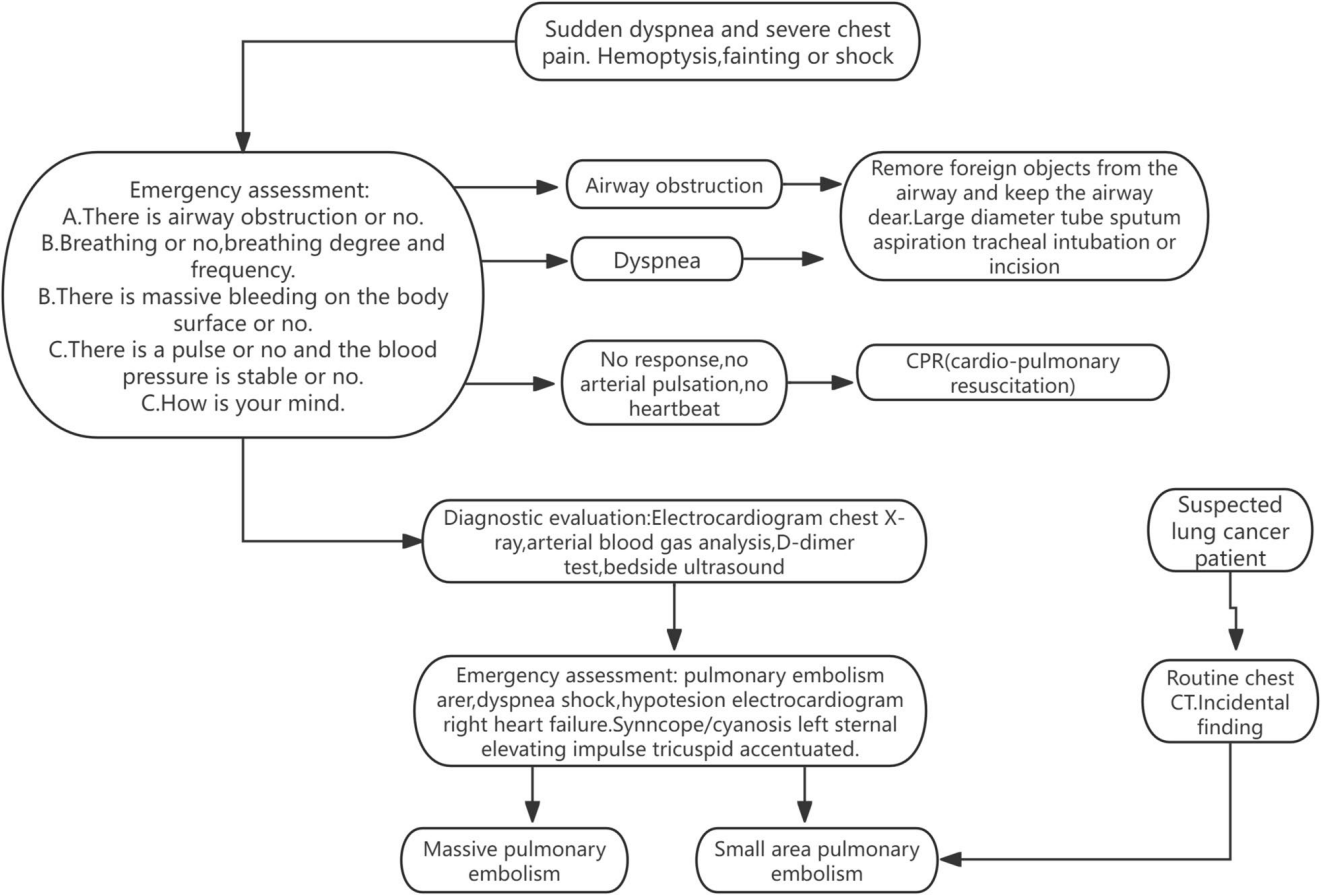
Key signs of pulmonary embolism and how to recognize it

The simultaneous diagnosis of lung cancer and PE introduces significant complexity to clinical management and decision-making. For example, individuals with operable early-stage lung cancer may face delays in treatment due to the need for surgical intervention. However, the administration of anticoagulant therapy poses a heightened risk of perioperative bleeding, complicating surgical management. As a result, the prognosis for individuals with concurrent lung cancer and PE is generally poorer compared to those with lung cancer alone. Figure 1 shows the main signs of pulmonary embolism and procedures for recognizing it.

## Risk factors and effects of anti-tumor therapies on PE in patients with lung cancer

Anti-tumor treatments, including surgery, chemotherapy, and immunotherapy, contribute to an increased risk of PE in individuals with lung cancer [17]. Various studies have identified intrinsic risk factors associated with lung cancer that predispose individuals to PE, such as elevated platelet counts, high D-dimer levels, hemoglobin concentrations exceeding 150 g/L, and reduced oxygen partial pressure below 70 mmHg [18]. In addition, adenocarcinoma is an independent risk factor for PE, and the risk of pulmonary embolism in patients with stage III-IV lung cancer is 2.97 times higher than that in patients with stage I-II [19]. Regarding genetics, among the 3 common lung cancer gene variants (EGFR mutation, KRAS mutation, and EML4/ALK rearrangement), EML4/ALK rearrangement is considered to be a high-risk factor for the development of PE [20]. However, the direct causal relationship between anti-tumor therapies and the development of PE remains insufficiently explored.

Surgical interventions in lung cancer are associated with a significantly elevated risk of thromboembolism, approximately threefold higher compared to non-surgical patients. This increased risk can be attributed to factors such as vascular endothelial damage during surgery, the release of tissue factors into the bloodstream, hypercoagulability, and postoperative immobility, which leads to blood stasis [21]. Song et al. reported that in patients undergoing lung cancer resection without preventive measures, the incidence of VTE could be as high as 16.40% [22], underscoring the substantial risk following surgery. Similarly, a retrospective analysis by Yang et al. involving 1,001 individuals with lung cancer found that the probabilities of developing VTE at 1, 3, 6, 12, and 30 months post-surgery were 2.0%, 3.0%, 4.0%, 5.0%, and 5.3%, respectively, with the first month being the peak period for VTE occurrence [23].

Chemotherapy significantly heightens the risk of thrombosis in individuals with lung cancer, increasing the likelihood by 2–6 times [21]. Chemotherapeutic agents such as cisplatin, carboplatin, gemcitabine, and paclitaxel have been shown to enhance procoagulant activity in endothelial cells by promoting the protein disulfide isomerase-dependent activation of tissue factor, resulting in direct endothelial damage and thrombus formation [24]. These drugs also stimulate the release of interleukin-1, which increases endothelial cell reactivity to platelets and upregulates the expression of adhesion molecules on the endothelial surface. Prolonged chemotherapy and radiotherapy can lead to hepatotoxicity, reducing natural anticoagulants and causing cumulative vascular endothelial damage, thereby increasing the risk of thrombosis over time.

In individuals with lung adenocarcinoma, epidermal growth factor receptor (EGFR) gene mutations are common, and EGFR-tyrosine kinase inhibitors (EGFR-TKIs), a class of targeted molecular therapies, are widely used in clinical practice. EGFR-TKIs are associated with an elevated risk of thrombosis due to their ability to activate platelets, promoting adhesion, aggregation, and release, thereby contributing to thrombotic events [23]. A report noted that three individuals with EGFR mutations developed PE while receiving first-line treatment with the third-generation EGFR-TKI, Osimertinib, at 6.1, 7.7, and 11.4 months, respectively [25].

Anti-angiogenic agents, such as bevacizumab, also present a risk of inducing PE by interacting with tumor-associated endothelial cells and shifting the endothelial state from anticoagulant to prothrombotic [26]. However, specific reports regarding this mechanism are limited.

The relationship between immunotherapy and the occurrence of PE remains unclear. Some studies have reported similar rates of PE development in individuals undergoing immunotherapy and those receiving chemotherapy. [27, 28] Nonetheless, the paucity of data highlights the need for further research to clarify this association.

Determining the optimal management of PE in individuals with lung cancer, whether by modifying the anti-tumor treatment regimen or continuing treatment alongside anticoagulant therapy, requires additional investigation. A comprehensive body of case studies and research is necessary to guide effective clinical decision-making.

## Incidental and symptomatic PE in lung cancer: diagnostic and clinical implications

The increasing use of chest CT scans for cancer staging, treatment evaluation, and recurrence assessment has led to a rise in the detection of both incidental pulmonary embolism (IPE) and symptomatic pulmonary embolism (SPE) in individuals with lung cancer [29].

In cases of concurrent lung cancer and PE, there is a possibility that IPE may progress to SPE during follow-up assessments, particularly following anti-tumor treatment. For individuals with lung cancer who exhibit pre-existing symptoms, the onset or worsening of respiratory distress, chest pain, hemoptysis, anxiety, decreased oxygen saturation, and other clinical signs should prompt consideration of both tumor progression and the presence of SPE.

Misdiagnosis or underdiagnosis of PE remains common in clinical settings due to its often subtle and nonspecific symptoms. The likelihood of PE varies across different lung cancer types, with adenocarcinoma having the highest incidence. In the study by Cui et al., 36.4% of individuals with lung cancer had PE, including 25 cases of lung adenocarcinoma, 3 cases of squamous cell carcinoma, 4 cases of pulmonary artery sarcoma, and 5 cases of unknown histology [8]. Recently, 20 cases of lung cancer combined with PE were reported in our center, consisting of 15 adenocarcinoma cases, 1 mucinous adenocarcinoma case, 1 squamous adenocarcinoma case, 2 cases of squamous cell carcinoma, and 1 case of small cell lung cancer. Adenocarcinoma is recognized as an independent risk factor for the development of PE [30].

The complexity of treating lung adenocarcinoma is well-established, and the concurrent occurrence of PE further complicates clinical management. Li et al. reported that the in-hospital mortality rate for individuals with malignancies and PE was 14.1%, compared to 6.1% for those with PE alone [31]. During lung cancer treatment, the risk of exacerbating or advancing pre-existing PE is significant. Therefore, careful attention to the coexistence of PE is essential in both the management and follow-up of individuals with lung cancer.

## Prevalence and prognostic implications of PE and pleural effusion in patients with lung cancer

Pleural effusion is notably common in individuals with PE, especially when complicated by pneumonia. Some studies have identified pleural effusion as a risk factor associated with poorer prognosis in individuals diagnosed with PE [32]. Among individuals with concurrent lung cancer and PE, 19 cases (47.5%) were found to have pleural effusion, compared to 13 cases (26%) in a control group, with the difference being statistically significant. This association may be linked to the underlying lung cancer, as pleural involvement and metastasis are common in advanced stages of the disease. Patients with advanced lung cancer frequently undergo treatments such as radiotherapy and immunotherapy, which can weaken their overall condition, increasing susceptibility to secondary infections.

Although the exact mechanisms behind this relationship require further clinical investigation, the appearance of new pleural effusion on chest imaging, combined with elevated D-dimer levels and clinical symptoms such as chest tightness and dyspnea, should raise suspicion of SPE in individuals with lung cancer [21, 33].

During the treatment of lung cancer, PE may develop secondary to pulmonary infections or as a result of disease progression in advanced-stage cancer. Differentiating between these conditions relies on imaging findings, where longitudinal tracking of changes in lesions over time provides more valuable information than single cross-sectional imaging studies. The sequence of symptom onset is also key in determining causality, supported by clinical indicators such as fever, dyspnea, airway secretions, and laboratory results. In most cases, pathological examinations are not necessary for distinguishing between these conditions.

## Post-pulmonary embolism syndrome in patients with lung cancer: prevalence and clinical implications

Post-pulmonary embolism (post-PE) syndrome, first introduced by Klok et al., refers to a clinical condition characterized by persistent, unexplained dyspnea, reduced physical activity, and impaired quality of life lasting for more than three months after an acute PE [34]. This syndrome is associated with abnormalities in pulmonary artery hemodynamics, right ventricular function, and gas exchange, both at rest and during physical activity.

With advancements in minimally invasive surgery for lung nodules, early detection of lung cancer, the development of novel therapeutic agents, and prolonged treatment regimens, survival rates for individuals with lung cancer are expected to improve. However, only about 15% of individuals with lung cancer are eligible for surgery, and postoperative outcomes vary. Risk factors such as chronic obstructive pulmonary disease (COPD), high CAT scores, and the type of surgery performed are linked to the development of postoperative respiratory failure [35].

In individuals with both lung cancer and concurrent PE, the combination of anticoagulant therapy and targeted oncological treatments may contribute to a sustained thrombotic burden. This could potentially lead to an increased incidence of chronic thromboembolic pulmonary disease, which is frequently associated with pulmonary arterial hypertension. The potential development of post-PE syndrome in individuals with lung cancer who have experienced PE requires further investigation, as its prevalence and clinical implications in this population have not been fully established.

## Management of venous thromboembolism in lung cancer: risks and therapeutic approaches

European guidelines recommend thrombolysis for clinically unstable individuals presenting with sub-massive or massive PE at admission, provided no contraindications exist [36]. For individuals without sub-massive or massive PE, anticoagulant therapy is the standard treatment, typically starting with subcutaneous low-molecular-weight heparin (LMWH) followed by a transition to direct oral anticoagulants (DOACs) [37]. Compared with the general population, individuals with cancer have an increased risk of recurrent VTE and bleeding complications related to anticoagulant therapy [38].

Research suggests that while the prophylactic use of anticoagulants in individuals with lung cancer does not improve overall prognosis, it increases the likelihood of hemorrhagic events. Therefore, anticoagulant therapy in this population requires a personalized approach, taking into account individual risk profiles and clinical conditions [39].

In addition to standard risk factors, other considerations such as adenocarcinoma subtype, genetic mutations, cancer stage, and the patient's medical history in the six months prior to chemotherapy initiation may offer further insights. These factors could guide clinical decisions regarding the potential benefit of prophylactic anticoagulation in select patient populations [40]. Studies have shown that the multidisciplinary team (MDT) model of lung cancer care not only improves the quality of life of patients, but also increases survival rates [41]. Despite limitations in the primary studies, the clinical implementation of MDTs seemed to improve outcomes of a patient with NSCLC, with a favorable risk–benefit ratio [42]. Given the coexistence of hypercoagulability and heightened hemorrhage risk during lung cancer treatment, the interaction between the individual's physiological state and prescribed medications must be carefully managed, as it can vary throughout different stages of treatment.

## Conclusion

The management of individuals with concurrent lung cancer and PE presents significant clinical challenges, requiring a comprehensive and individualized approach. Existing management strategies are not fully adequate and may lack the necessary rigor to address the complexities of these conditions. Ongoing research and interdisciplinary collaboration are crucial to advancing more effective and targeted treatment solutions for this high-risk patient population.

## Data Availability

Date will be made available on request from the corresponding author (Yi-Wen Zhang) on reasonable request.
